# Meanings, Mechanisms, and Measures of Holistic Processing

**DOI:** 10.3389/fpsyg.2012.00553

**Published:** 2012-12-12

**Authors:** Jennifer J. Richler, Thomas J. Palmeri, Isabel Gauthier

**Affiliations:** ^1^Department of Psychology, Vanderbilt UniversityNashville, TN, USA

**Keywords:** holistic processing, face recognition, face perception, face processing, object recognition

## Abstract

Few concepts are more central to the study of face recognition than holistic processing. Progress toward understanding holistic processing is challenging because the term “holistic” has many meanings, with different researchers addressing different mechanisms and favoring different measures. While in principle the use of different measures should provide converging evidence for a common theoretical construct, convergence has been slow to emerge. We explore why this is the case. One challenge is that “holistic processing” is often used to describe both a theoretical construct and a measured effect, which may not have a one-to-one mapping. Progress requires more than greater precision in terminology regarding different measures of holistic processing or different hypothesized mechanisms of holistic processing. Researchers also need to be explicit about what meaning of holistic processing they are investigating so that it is clear whether different researchers are describing the same phenomenon or not. Face recognition differs from object recognition, and not all meanings of holistic processing are equally suited to help us understand that important difference.

For years, holistic processing has been used to explain what makes face recognition special. Unfortunately, many studies provide only verbal descriptions of holistic processing and there is growing consensus that the concept is too loosely defined. A well-known limitation of verbal descriptions is that the same word can mean different things to different readers (e.g., Hintzman, 1990), and “holistic processing” is no exception. While there have been attempts to operationalize holistic processing mechanistically, using computational models defined mathematically or by simulation (Wenger and Townsend, 2001; Richler et al., 2008a; Fific and Townsend, 2010; Mack et al., 2011; Gold et al., 2012), this is just one important step.

Consider a recent paper by Gold et al. (2012). They compared performance matching individual face parts to performance matching whole faces. Contrast was staircased for the various parts and whole faces independently. An integration index was calculated as sensitivity for the whole face divided by the sum of sensitivities for parts. Based on an ideal observer model, an index greater than one indicates superoptimal integration – matching the whole face would be literally greater than the sum of its parts. No evidence for superoptimal integration was found. The authors concluded that faces are not processed holistically, contrary to many claims. The generality of this claim hinges on how holistic processing is defined. Gold et al.’s integration index is consistent with one proposed meaning of holistic processing – perceptual integration of parts into a unitary whole – but this may not be relevant to alternative meanings.

We have singled out Gold et al. (2012) for both its strengths and weaknesses. On the one hand, it exemplifies what we hope to see as a growing trend in the field: they developed and tested a precise computational definition of holistic processing. On the other hand, it highlights a recurring challenge. While the multiplicity of meanings of holistic processing is often acknowledged in reviews and theoretical articles (e.g., Gauthier and Tarr, 2002; Maurer et al., 2002; Kimchi and Amishav, 2010), a majority of papers only use one meaning of holistic processing but draw general conclusions that may erroneously span all possible meanings.

Here, we first discuss measures and mechanisms commonly associated with holistic processing. The term “holistic” sometimes refers to a theoretical position regarding mechanisms. Different proposed mechanisms can be “holistic” in different respects, yet the same term is used for all of them. Other times, “holistic” refers to some measured behavior within specific tasks. The same term is applied to different measures, even though they may be capturing different things. We next highlight the various potential meanings of holistic processing. We discuss the importance of being explicit about those meanings to ensure that we do not confuse different phenomena, independently of the proposed mechanisms that may ultimately explain them, and to guide predictions about which measures might be expected to converge.

## Multiple Measures and Mechanisms

A review of the literature reveals at least a dozen different tasks that ostensibly measure holistic processing of faces. The two most popular are the part-whole task and the composite task. In the part-whole task, holistic processing is measured as better recognition of a feature (e.g., eyes) when that feature is presented in the context of a whole face versus when it is presented in isolation (Tanaka and Farah, 1993). In the sequential matching composite task (Farah et al., 1998), participants judge whether one face half (e.g., top) of two sequentially presented faces is the same or different while ignoring the other face half (e.g., bottom). Holistic processing is measured as interference from the task-irrelevant part, which is attenuated by misalignment.

Some prominent debates revolve around whether or not versions of these various tasks are even appropriate for measuring holistic processing in the first place (e.g., partial versus complete composite task, Gauthier and Bukach, 2007 versus McKone and Robbins, 2007; part-whole task, Leder and Carbon, 2005). A more recent question has been whether these measures tap into the same construct by considering the correlation between performance on these tasks. Some have found a correlation (DeGutis et al., 2013). Others have not (Wang et al., 2012).

**Figure 1:**
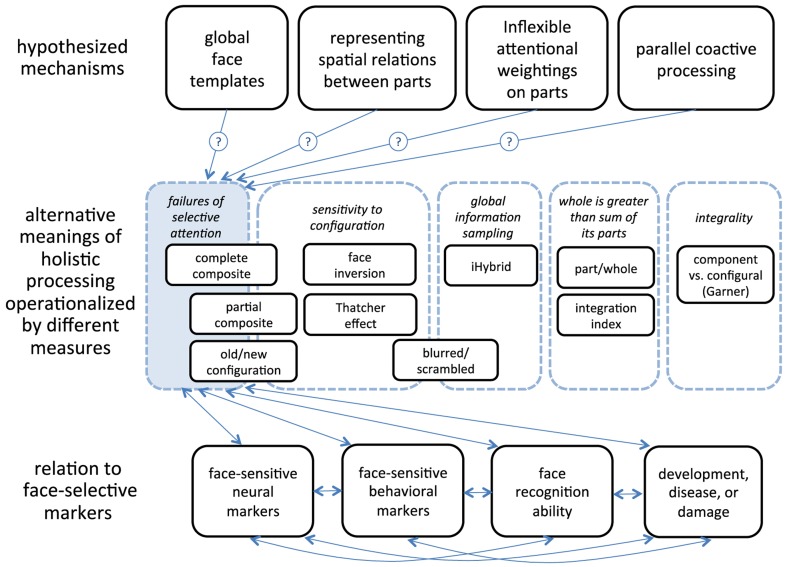
**Framework for approaching the study of holistic processing**. Tasks used to measure holistic processing cluster into different possible meanings. The partial design (Robbins and McKone, 2007) and complete design (Richler et al., 2008b) of the composite task and the old/new configuration task (Tanaka and Sengco, 1997) measure failures of selective attention and to some extent sensitivity to configuration; the Thatcher illusion (Thompson, 1980) and the face inversion effect (Yin, 1969) measure sensitivity to configuration; iHybrid (Miellet et al., 2011) measures the use of global information sampling; The blurred/scrambled task measures both global information sampling and sensitivity to configuration (Hayward et al., 2008); An integration index (Gold et al., 2012) and the Part/whole task (Tanaka and Farah, 1993) measure whether the whole is more than the sum of its parts; the component versus configural Garner paradigm (Amishav and Kimchi, 2010) measures integrality of features and configurations. These different meanings must be validated in terms of establishing relationships to face-selective markers. A given meanings of holistic processing may be driven by any of the hypothesized mechanisms.

One important goal of using and comparing measures of holistic processing is to test hypotheses about the mechanism(s) giving rise to holistic recognition. Numerous mechanisms have been proposed (Figure 1). The most commonly cited is that faces are recognized holistically because faces are represented as undifferentiated wholes. Face parts are “glued” together into a unitary perceptual representation or “face template” (e.g., Young et al., 1987; Tanaka and Farah, 1993; Farah et al., 1998; Maurer et al., 2002).

An alternative “configural” view is that representations about individual face parts and their configuration are used to recognize faces, with configural information particularly dominant for upright faces. Holistic processing then reflects explicit representation of spatial relations between features (e.g., Diamond and Carey, 1986; Rhodes et al., 1993; Searcy and Bartlett, 1996; Leder and Bruce, 2000). Kimchi and colleagues proposed that neither featural nor configural information dominates upright face perception, but that holistic processing emerges from interactive processing between the two (Amishav and Kimchi, 2010; Kimchi and Amishav, 2010). Fific and Townsend (2010) proposed that holistic processing reflects parallel coactive processing of multiple features and possibly their configurations.

Alternatively, holistic processing could reflect a perceptual strategy of processing all parts together that becomes automatized for categories for which people have extensive individuation experience, like faces (Wong and Gauthier, 2010; Richler et al., 2011c,d). Although independent part representations are available, expertise with faces engenders limited flexibility in weighting face parts during experimental tasks that require participants to treat faces unnaturally, as when instructed to ignore one face part.

It remains unclear how these various proposed mechanisms and measures are related. It could be that all, some, or none of these holistic mechanisms explain any given combination of holistic measures.

## Multiple Meanings

Researchers debate which measures best capture holistic processing and what mechanisms best predict those measures. We anticipate continued stalemate unless researchers recognize that there are different *meanings* of holistic processing and are explicit about the particular meaning they are investigating.

Different measures of holistic processing cluster into different categories that reflect different aspects of what it might mean for face recognition to be “holistic” (Figure 1). Some measures overlap with multiple meanings. For example, the composite task illustrates a failure to selectively attend to a face part – one empirical signature of holistic face recognition – but alignment of parts is often manipulated in this task, tapping into another empirical signature, sensitivity to configuration.

Having multiple measures for a single theoretical construct is important to establish convergent validity. But convergence between measures is not expected if they map onto different meanings. By itself, this statement seems obvious. But we often see arguments that two measures should be related simply because they are both tagged with the term “holistic processing,” without considering that there could be divergent meanings to the term “holistic.”

For example, consider research comparing the part-whole task and the composite task (Wang et al., 2012; DeGutis et al., 2013). Although both tasks ostensibly measure “holistic processing,” it is not clear how they relate to one another: in the part-whole task, holistic processing is reflected by an advantage making judgments of the whole compared to individual parts; participants are never asked to ignore part of a face. In the composite task, participants must selectively judge one part while actively ignoring another part. It is logically possible to perform better when a whole face context is available (whole-part advantage), while also ignoring that face context if instructed to do so (no composite effect). These tasks may tap into two different possible meanings of holistic processing – the whole is greater than the sum of its parts versus a failure of selective attention. Performance on these tasks could be correlated if they are driven by the same underlying mechanism, which may or may not be the case.

Conversely, we might expect correlations between tasks that are grouped together under the same meaning. For example, we might expect a correlation between the face inversion effect and the Thatcher effect if they both measure sensitivity to configuration.

Figure 1 illustrates a number of possible meanings of holistic processing. We can ask whether there are costs to selectively attending to one face part, whether configural cues in faces are particularly important, or whether a whole face is greater than the sum of its parts. But we also need to ask these questions about *non-face objects*. After all, understanding how face recognition and object recognition differ – what makes faces “special” – is often what motivated researchers to study holistic processing in the first place (e.g., Tanaka and Farah, 1993).

It is not uncommon for purported measures of holistic processing to be applied to faces but not objects; this includes the integration index of Gold et al. (2012), component versus configuration variant of the Garner paradigm (Amishav and Kimchi, 2010), and the recent iHybrid technique (Miellet et al., 2011). Without testing these measures on non-face objects, it remains unknown whether the meanings of holistic processing they tap into reflect what is special to face recognition or more general properties of object recognition.

For example, consider the Thatcher Illusion (Thompson, 1980), where it is remarkably difficult to detect whether local features (e.g., eyes) have been inverted when the entire face is inverted. When that same face is presented upright, the inverted local features are obvious – the face looks hideous. Apart from being an engaging demo for an introductory psychology course, interest in the Thatcher illusion partly centers on its seeming face-specific nature. Yet the illusion for faces is not exceptionally large compared to the distribution of analogous effects obtained across many non-face categories (Wong et al., 2010).

In the process of comparing faces to objects, it is important to recognize that people differ markedly on their ability to recognize different categories of non-face objects. The distribution of the Thatcher illusion observed with non-face objects is just a manifestation of a general principle: finding that face and object recognition are associated or dissociated visual abilities depends on what object categories are contrasted with faces (McGugin et al., 2012). Studies finding larger composite effects for faces than objects suffer from the same limitation: only one non-face object category is used (e.g., Cassia et al., 2009).

The Thatcher illusion is also only one of many face measures that compare upright versus inverted face recognition. A classic finding is that inversion disproportionately disrupts face recognition compared to recognition of other objects (Yin, 1969). This *quantitative* difference between upright and inverted face has sometimes been taken as evidence for a *qualitative* difference – upright faces are recognized holistically and inverted faces are not. But Richler et al. (2011c) found that both upright and inverted faces are processed holistically, based on meaning of holistic processing grounded in a failure of selective attention measured using the complete design composite task. This finding has led some to suggest that this meaning and measure must not reflect “true” holistic processing because they do not reveal a qualitative difference between upright and inverted faces (e.g., Palermo et al., 2011). This, however, presumes that the difference between upright and inverted face recognition must be qualitative. Importantly, Richler et al. did find that upright and inverted faces differ in holistic processing when processing time is considered, indicative of a quantitative difference between upright and inverted face recognition.

Besides differences between faces and objects or upright and inverted faces, other criteria can help assess meanings of holistic processing. For example, to what degree are certain meanings of holistic processing related to face-selective markers like FFA activity (Weiner and Grill-Spector, 2011) or N170 modulation (Bentin et al., 1996)? How does holistic processing vary with performance on the Cambridge Face Memory Task (Duchaine and Nakayama, 2006), increasingly considered a gold standard for measuring individual differences in face recognition (Wilmer et al., 2010; Germine et al., 2011)? If holistic processing makes face recognition special, as suggested since the earliest mentions of holism in the context of faces, then it might be related to modulation of brain activity in face-specific regions and variability in face recognition abilities. Considering these relationships harkens to classic discussions of establishing construct validity by connecting theoretical constructs with empirical measures (Cronbach and Meehl, 1955).

## Holistic Processing as a Failure of Selective Attention

Understanding a concept like holistic processing demands maintaining distinctions and establishing connections at various levels. We illustrate this now with reference to some of our own work on holistic processing that uses the complete design composite task to test a meaning of holistic processing as a failure of selective attention. This measure has good construct validity: it captures differences between objects and faces (Farah et al., 1998; Richler et al., 2011c), correlates with face-selective neural markers (Gauthier and Tarr, 2002; Gauthier et al., 2003; Wong et al., 2009b), and predicts face recognition performance (Richler et al., 2011a; McGugin et al., 2012; DeGutis et al., 2013). Furthermore, it suggests possible origins of holistic face processing^1^, since these failures of selective attention increase with expertise with non-face objects (Wong et al., 2009a; Bukach et al., 2010; Boggan et al., 2012).

Other seemingly similar measures may not meet these criteria. For example, holistic processing measured with the partial design composite task (e.g., Michel et al., 2006; Wang et al., 2012) is modulated by manipulations that theoretically should influence response bias but not holistic processing, such as deceptive instructions regarding the proportion of “same” trials (Richler et al., 2011b). This suggests that the partial design may have less value in efforts to relate individual differences in markers of holistic processing to one another (Wang et al., 2012) or to neural markers (Jacques and Rossion, 2009; Schiltz et al., 2010) since it does not capture a putatively stable ability.

Behaviorally, holistic processing within the composite task is characterized as a failure of selective attention. But it is critical to recognize that meanings like “failures of selective attention,” “sensitivity to configuration,” or “the whole is greater than the sum of its parts” do not map one-to-one onto possible mechanisms. Many diverse empirical findings could emerge because faces are represented in terms of global face templates and/or because face recognition engenders inflexible attentional weightings.

For example, while it can be alluring to directly map performance in the partial design composite task onto global face templates (e.g., Young et al., 1987; Robbins and McKone, 2007) and behavior in the complete design composite task onto inflexible attentional weightings, we believe that making behavior-mechanism mappings requires a combination of computational modeling and converging empirical evidence. It is premature to infer a particular mechanism only on the basis of observing a particular behavioral effect (e.g., Michel et al., 2006, 2007; Ramon et al., 2010; Palermo et al., 2011), without testing whether that mechanism actually predicts that observed behavior. The language used to describe empirical effects needs to remain distinct from the language used to describe mechanisms. The composite effect on its own does not speak to any particular underlying mechanism, regardless of how it is verbally described.

Indeed, models that assume that faces are represented by relatively holistic, template-like representations (Cottrell et al., 2002; see Palmeri and Cottrell, 2009) and models that assume that faces are represented by orthogonal dimensions representing face parts can both predict behavior in the composite task (Richler et al., 2007). Furthermore, behaviors that seem to suggest a mechanistic model assuming a decisional locus of holistic effects (Wenger and Ingvalson, 2002; Richler et al., 2008a) can also be mirrored by a mechanistic model assuming a perceptual locus, rendering results about perceptual versus decisional loci inconclusive (Mack et al., 2011; Silbert and Thomas, 2012). So the mapping between measures and mechanisms is not necessarily one-to-one (see also Ross et al., in preparation), even when language suggests otherwise.

Failure of selective attention in face recognition can arise mechanistically because of the representational constraints of a global face template or because of inflexibility in attentional weightings on face parts. We favor the latter based on emerging empirical evidence. For example, when face and novel object composites are interleaved, novel objects are processed more holistically when preceded by an aligned face, which is processed holistically, than a misaligned face, which is not (Richler et al., 2009). This is difficult to explain by a face template. How would a unified perceptual representation of an aligned face impact processing of a subsequent novel object that does not share the same configuration of features? These results seem more consistent with holistic processing being a consequence of a perceptual strategy that is automatically recruited for the aligned face stimulus and remains in play when a novel object is processed. Other studies suggest that failures of selective attention in the composite task can be influenced by inducing global or local processing biases (Gao et al., 2011; Curby et al., 2012), hinting that holistic processing could be the outcome of an attentional strategy. While it is not clear mechanistically how these effects emerge, it is premature to equate holistic processing with holistic representations.

## Conclusion

Holistic processing can mean many different things, and all meanings are not created equal: some may not distinguish face and object processing, predict neural markers or individual differences, or manifest themselves in behavior despite being reasonable definitions for how face processing could be “holistic.” At present, holistic processing as a failure of selective attention seems the strongest candidate. Certainly holistic processing may be conceptualized in other ways, and better measures and mechanistic models may be developed. Currently, other meanings lack critical evidence, in many cases simply because these relationships have not yet been tested. Multiple meanings of holistic processing may turn out to be valid, and several may eventually merge into an overarching construct on the basis of empirical studies and computational modeling. Using “holistic processing” as a blanket term for all possible meanings is a challenge for the field, leading researchers to expect that a variety of markers of holistic processing should all be related when they may not measure the same construct and may be driven by different mechanisms. We cannot change the words people use, but we urge people to recognize the different ways holistic processing can refer to different meanings, mechanisms, and measures.

## Conflict of Interest Statement

The authors declare that the research was conducted in the absence of any commercial or financial relationships that could be construed as a potential conflict of interest.

## Footnotes

^1^Note that to date developmental studies examining composite effects in children have only used the partial design composite task (e.g., Mondloch et al., 2007; Cassia et al., 2009).
